# An 18-Gene Signature for Vascular Invasion Is Associated with Aggressive Features and Reduced Survival in Breast Cancer

**DOI:** 10.1371/journal.pone.0098787

**Published:** 2014-06-06

**Authors:** Monica Mannelqvist, Elisabeth Wik, Ingunn M. Stefansson, Lars A. Akslen

**Affiliations:** 1 Centre for Cancer Biomarkers CCBIO, Department of Clinical Medicine, University of Bergen, Norway; 2 Department of Pathology, The Gade Institute, Haukeland University Hospital, Bergen, Norway; Rutgers - New Jersey Medical School, United States of America

## Abstract

**Aims:**

Vascular invasion by tumor cells is known to be important for cancer progression. By microarray and qPCR analyses, we earlier identified an 18-gene signature associated with vascular involvement in endometrial cancer. Here, we explored the significance of this vascular invasion signature in multiple series of breast cancer patients.

**Methods and Results:**

The study includes 11 open access gene expression data sets which collectively provide information on 2423 breast cancer patients. The 18-gene signature showed consistent associations with aggressive features of breast cancer, like high tumor grade, hormone receptor negativity, HER2 positivity, a basal-like phenotype, reduced patient survival, and response to neoadjuvant chemotherapy. Also, the vascular invasion signature was associated with several other gene expression profiles related to vascular biology and tumor progression, including the Oncotype DX breast cancer recurrence signature.

**Conclusions:**

The 18-gene vascular invasion signature showed strong and consistent associations with aggressive features of breast cancer and reduced survival.

## Introduction

Vascular invasion, *i.e.* tumor cells entering the vascular system, is considered to be an early step in the metastatic process and important for the progress of malignant tumors. When examined on tissue sections as a morphologic marker, the presence of vascular invasion is a strong prognostic factor in breast cancer and other tumor types [1]–[4]. Recently, we presented a gene expression signature related to vascular invasion in endometrial cancer, being associated with aggressive tumor features and reduced survival [5]. This signature was generated from 57 primary endometrial tumors, and the gene expression pattern was investigated by microarray and qPCR, and subsequently related to the presence of vascular invasion on tissue sections. Finally, 18 significantly and differentially expressed genes were found between tumors with and without such vascular involvement. Here, we explored whether this 18-gene vascular invasion signature was associated with high-grade features and poor survival in breast cancer, and we examined a broad panel of publicly available data sets, collectively representing a total of 2423 patients. The signature genes were investigated in these external data sets and related to clinical data and follow-up information. Briefly, the vascular invasion signature was associated with markers of aggressive breast cancers and reduced survival, and the vascular invasion score was also associated with other published gene signatures related to vascular involvement and tumor progression.

## Materials and Methods

### Vascular invasion signature

Generation of the 18-gene vascular invasion gene expression signature was originally identified in a prospectively collected patient series of 57 endometrial carcinomas by microarray and qPCR analysis [5]. The vascular invasion signature consists of 7 up-regulated and 11 down-regulated genes (**Table 1**). The vascular invasion signature was based on supervised analyses of gene expression differences related to lymphatic and blood vessel involvement (assessed on HE-sections) [5], and the signature showed significant association with patient survival and aggressive clinico-pathologic features, as well as with vascular and matrix biology.

**Table 1:** The vascular invasion signature consists of 7 up-regulated and 11 down-regulated genes [5].

Gene symbol	Gene name
Up-regulated genes	
MMP3	Matrix metallopeptidase 3 (stromelysin 1, progelatinase)
TNFAIP6	Tumor necrosis factor, alpha-induced protein 6
FPR2	Formyl peptide receptor 2
IL8	Interleukin 8
ANGPTL4	Angiopoietin-like 4
SERPINE1	Serpin peptidase inhibitor, clade E (nexin, plasminogen activator inhibitor type 1), member 1
COL8A1	Collagen, type VIII, alpha 1
Down-regulated genes	
OGN	Osteoglycin
ATCAY	Ataxia, cerebellar, Cayman type
MAMDC2	MAM domain containing 2
COL4A6	Collagen, type IV, alpha 6
C1orf114	Chromosome 1 open reading frame 114
KLHL13	Kelch-like 13 (Drosophila)
OSR2	Odd-skipped related 2 (Drosophila)
ALDH1A2	Aldehyde dehydrogenase 1 family, member A2
SEMA5A	Sema domain, seven thrombospondin repeats (type 1 and type 1-like), transmembrane domain (TM) and short cytoplasmic domain, (semaphorin) 5A
FGFR2	Fibroblast growth factor receptor 2
ITIH5	Inter-alpha (globulin) inhibitor H5

### Gene expression data sets

Publicly available data sets with clinical information on breast cancer patients were found and downloaded from the Gene Expression Omnibus (GEO) website (www.ncbi.nlm.nih.gov/geo). Overall, 11 breast cancer data sets with clinical information were identified and studied, including a total of 2423 patients. Gene expression data from the following cohorts were analyzed:

**GSE1456**. A population based breast cancer series from 159 tumors with clinical information on histologic tumor grade, molecular tumor subclasses (as described by Sørlie et al. [6]), recurrence free survival, and breast cancer specific deaths [7].

**GSE20271**. Gene expression data on 178 breast cancer patients, clinical stage I–III, from 6 different international sites with data on histologic grade, estrogen receptor (ER), progesterone receptor (PR) and human epidermal growth factor receptor 2 (HER2) status [8].

**GSE20194**. 230 stage I–III breast cancers from fine-needle aspiration specimens before any therapy, with data on histologic grade, ER, PR, and HER2-status [9].

**GSE5460**. 129 primary, untreated breast cancers, balanced for nodal status, with information on tumor type and tumor size, histologic grade, lymphatic vascular invasion (LVI), ER-status, HER2-status, and lymph node status [10].

**GSE7849**. 78 tumors from women with early stage breast cancer with information on histological type, nuclear grade, LVI, ER-status, PR-status, lymph node status, and recurrence free survival [11].

**GSE20685**. 327 primary breast cancers with data on molecular subtypes, recurrence free survival and overall survival. The molecular subtypes were classified in I-VI, where subtypes I and II correspond to the basal-like and HER2 subtypes, subtype III represents a mixture of HER2 and Luminal B, subtype IV is similar to Luminal B, and subtype V and VI correspond to Luminal A tumors [12].

**GSE26639**. 226 primary breast carcinomas in stage II–III, with data on histologic grade, ER-status, PR-status, and HER2-status [13].

**GSE25066**. 508 HER2-negative breast cancers with data on tumor subclasses, ER-status, PR-status, and distant relapse free survival [14].

**GSE22358**. 154 stage II–III breast cancers with data on histologic grade, molecular subtype, ER-status, PR-status, HER2-status, p53 status and response to treatment [15].

**GSE17705**. 298 ER-positive breast cancers treated with tamoxifen for 5 years with data for distant relapse free survival [16].

**GSE12093**. 136 ER-positive breast cancer patients treated with tamoxifen with data on disease free survival [17].

### Gene expression signatures related to tumor progression

We used the following published gene expression signatures to investigate a possible correlation with the 18-gene vascular invasion score: The *VEGF signature* identifies a compact *in vivo* hypoxia signature highly expressed in metastatic breast tumors. This signature is associated with poor outcome in multiple tumor types [18]. *Wound response signature*; cancer invasion and metastasis have demonstrated similarities with the wound healing process. A published wound response signature predicts increased risk of metastasis and death in several cancers [19]. *NF-κB-regulated genes* are involved in tumor progression like proliferation, invasiveness, angiogenesis, lymphangiogenesis and inflammation. The NF-κB-associated gene signature contains 60 genes and is known to be of importance for tumor progression in inflammatory breast cancer [20]. *Hypoxia gene signature*; tumor hypoxia is an important feature of human cancer progression. This published hypoxia gene signature has demonstrated prognostic importance in breast and ovary cancers [21]. *BMI-1 driven gene signature*; BMI-1 participates in determining the proliferative potential and is required for self-renewal of different stem cells. The BMI-1 driven gene signature shows prognostic impact in many cancers [22]. Tumor stem cells and stemness features are important for tumor progression [23]. *Oncotype DX Recurrence Score* corresponds to the likelihood of breast cancer recurrence. The signature includes 5 reference and 16 cancer related genes [24].

### Gene expression signature scores

The genes from the vascular invasion signature and the other signatures, used for correlation studies, were mapped to the breast cancer microarray data sets. A few genes in some of the signatures could not be mapped to some of the data sets. Signature gene expression scores were generated according to the algorithms applied in the papers publishing the specific signatures. For the vascular invasion signature, the hypoxia signature and the BMI-1 driven signature, summarized expression values for the down-regulated genes were subtracted from the sum of expression values for the up-regulated genes. For the wound response signature, a summary expression signature was generated for the activated genes. For the VEGF signature and the NF-κB-regulated genes, a mean expression value from the expression values for the genes in the signature was calculated. For the Oncotype DX recurrence score, the algorithm in the paper was used on the 16 cancer-related genes.

### Statistics

Statistical analyses were performed with the PASW statistical software package version 17 (SPSS Inc., Chicago, IL). Correlations between categorical and continuous variables were assessed by non-parametric tests; Mann-Whitney (two categorical groups) or Kruskal-Wallis (>2 categorical groups) with a significance level of 0.05. Spearman's correlation (rho) was also calculated between tumor grade and the vascular invasion score. Linear association between two continuous variables was evaluated by linear regression analysis and Spearman's correlation. Univariate survival analyses were performed using the Kaplan-Meier method (log-rank significance test), and scores were dichotomized based on the upper quartile. Signature scores, together with standard clinico-pathological and molecular variables, were further analyzed by log-log plot to determine how these variables could be incorporated in Cox' proportional hazards regression model, and tested by the backward stepwise likelihood ratio test.

## Results

### Correlations to histologic grade and lymphatic vascular invasion

Seven of the data sets had information on histologic or nuclear tumor grade [7]–[10], [13]–[15], and all sets showed significant correlations between high signature score and high tumor grade (**Table 2**).

**Table 2:** Associations between histologic grade and the 18-gene vascular invasion signature score (mean signature score is given).

	Grade				
	1	2	3	p-value^1^	Correlation^6^
GSE25066 (N = 508)	−2.04	−1.31	1.09	<0.001	0.27
GSE22358 (N = 154)	−1.55	−0.82	2.27	0.001	0.31
GSE26639^2^ (N = 226)	−1.81	−1.11	1.06	0.001	0.25
GSE1456^3^ (N = 159)	−1.23	−1.03	1.32	<0.001	0.25
GSE20271^4^ (N = 178)	−2.09	−0.69	0.24	0.006	0.24
GSE 20194^4^ (N = 230)	−2.70	−1.21	1.24	<0.001	0.33
GSE5460^5^ (N = 129)	−2.57	−0.86	1.39	0.001	0.25

1 Kruskal-Wallis test, significance level 0.05,

2 Histologic grade,

3 Elston & Ellis tumor grade,

4 Modified Black's nuclear grade,

5 Modified Bloom–Richardson grade,

6 Spearman's rho.

Two data sets had information on lymphatic vascular invasion (LVI), but there was no significant direct correlation between LVI and the 18-gene signature score (data not shown) [10], [11]. As indicated, the original vascular invasion signature was based on vascular invasion as a combination of lymphatic and blood vessel involvement assessed on HE-sections [5].

### Increased vascular invasion score is associated with hormone receptor negative tumors

Seven of the data sets had information on ER-status [8]–[11], [13]–[15], and six of the sets showed significant correlations between high signature score and ER-negative tumors, five of them highly significant (p<0.001). The seventh data set did not show a significant correlation (**Table 3**). For PR-status, there was information available in six of the data sets [8], [9], [11], [13]–[15]. All sets showed a significant association between high signature score and PR-negative tumors (**Table 3**).

**Table 3:** Associations between ER-status, PR-status, HER2-status and the 18-gene vascular invasion signature score (mean signature score is given).

	ER			PR			HER2		
	Neg	Pos	p-value^1^	Neg	Pos	p-value^1^	Neg	Pos	p-value^1^
GSE22358 (N = 154)	2.53	−1.44	<0.001	1.27	−1.14	0.015	−0.12	2.21	0.041
GSE26639 (N = 226)	1.71	−1.12	<0.001	1.12	−1.52	<0.001	−0.41	0.68	NS
GSE20271 (N = 178)	0.52	−0.52	0.045	0.58	−0.77	0.006	−0.08	0.12	NS
GSE 20194 (N = 230)	1.87	−1.15	<0.001	0.84	−0.98	0.003	−0.19	1.00	0.071
GSE25066 (N = 508)	1.54	−1.08	<0.001	1.11	−1.19	<0.001	-	-	-
GSE7849 (N = 78)	0.75	−0.52	NS	0.89	−0.79	0.038	-	-	-
GSE5460 (N = 129)	2.07	−1.44	<0.001	-	-	-	−0.59	1.87	0.016

1 Mann-Whitney U test, significance level 0.05.

Five data sets contained information about HER2-status [8]–[10], [13], [15]. Two of them showed a significant correlation between high signature score and HER2-positive tumors, one data set had a borderline significant association, and two data sets did not show any significant association between the signature score and HER2-status (**Table 3**).

### Increased vascular invasion score is associated with molecular subtypes of breast cancer

**Figure 1:**
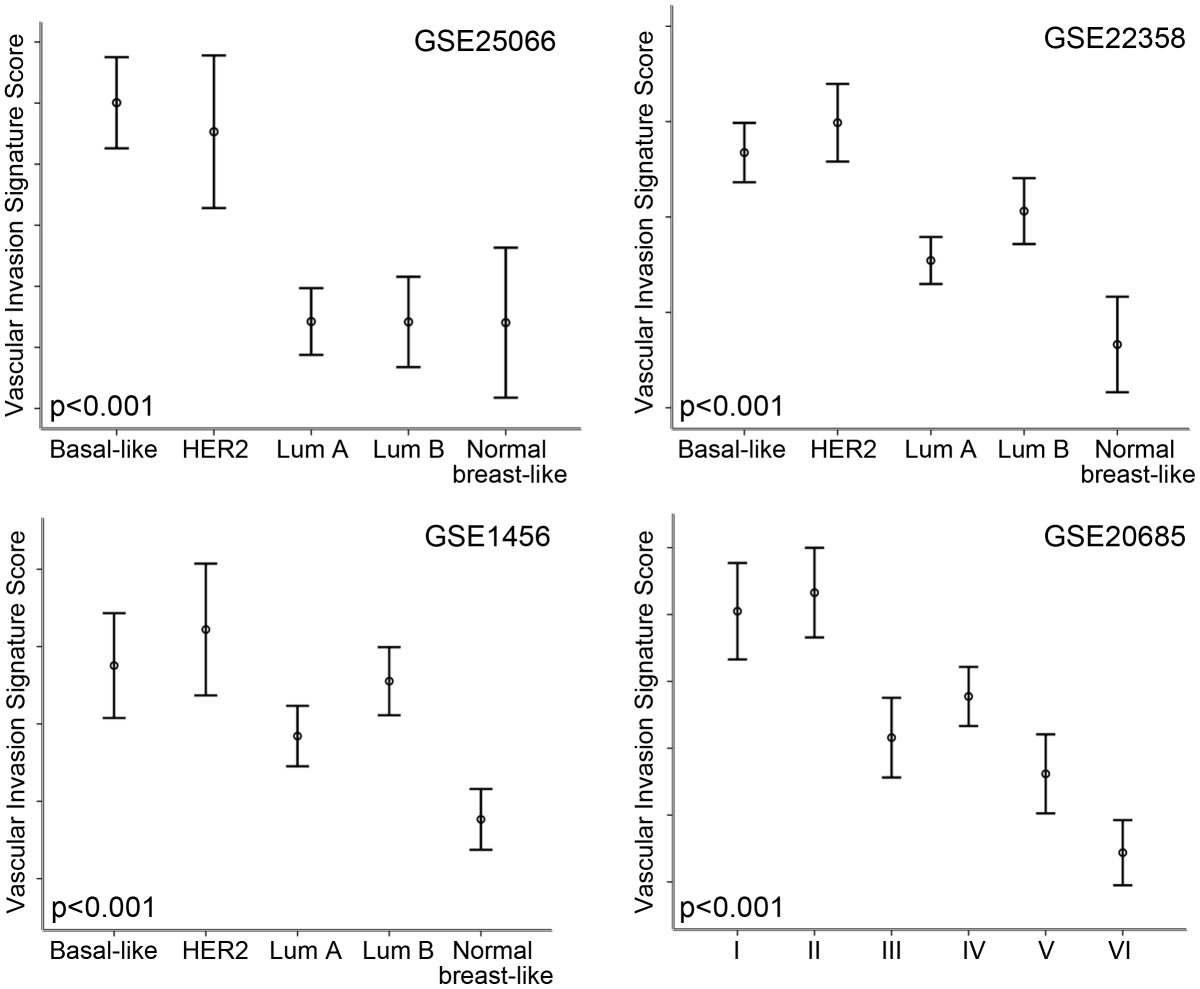
High Vascular Invasion Signature score is associated with Basal-like and HER2 molecular subtypes. High signature score is associated with Basal-like and HER2 molecular subtypes among data sets GSE25066, GSE22358, GSE1456 and GSE20685. Correlations were assessed by Kruskal-Wallis test. Mean expression signature scores indicated by circles, and the bars represent standard error ±2.

Three of the data sets had information on molecular subtypes of breast cancer such as Luminal A, Luminal B, HER2, basal-like, and Normal breast-like [7], [14], [15]. All data sets showed highly significant correlations between the subtypes and the vascular invasion signature score, p<0.001 (**Table 4**). The most aggressive basal-like and HER2 subtypes showed the highest signature score and Luminal A, Luminal B and normal breast-like the lowest. A fourth data set had molecular subtypes classified from I–VI [12]. When compared with the Sørlie classification [6], the results are similar to the three data sets mentioned above (**Table 4** **and** **Figure 1**).

**Table 4:** Associations between breast cancer molecular subtypes and the 18-gene vascular invasion signature score (mean signature score is given).

	Molecular subtypes						
	Basal-like	ERBB2	Luminal A	Luminal B	Normal breast-like		p-value^1^
GSE25066 (N = 508)	2.01	1.53	−1.58	−1.58	−1.60		<0.001
GSE22358 (N = 154)	3.38	4.94	−2.28	0.31	−6.68		<0.001
GSE1456 (N = 159)	1.89	3.05	−0.39	1.38	−3.09		<0.001
GSE 20685^2^ (N = 327)	5.12^I^	5.81^II^	0.40^III^	1.94^IV^	−0.96^V^	−3.91^VI^	<0.001

1 Kruskal-Wallis test, significance level 0.05,

2 Molecular subtypes I–VI [12].

### Increased vascular invasion score is associated with reduced overall and recurrence free survival

**Figure 2:**
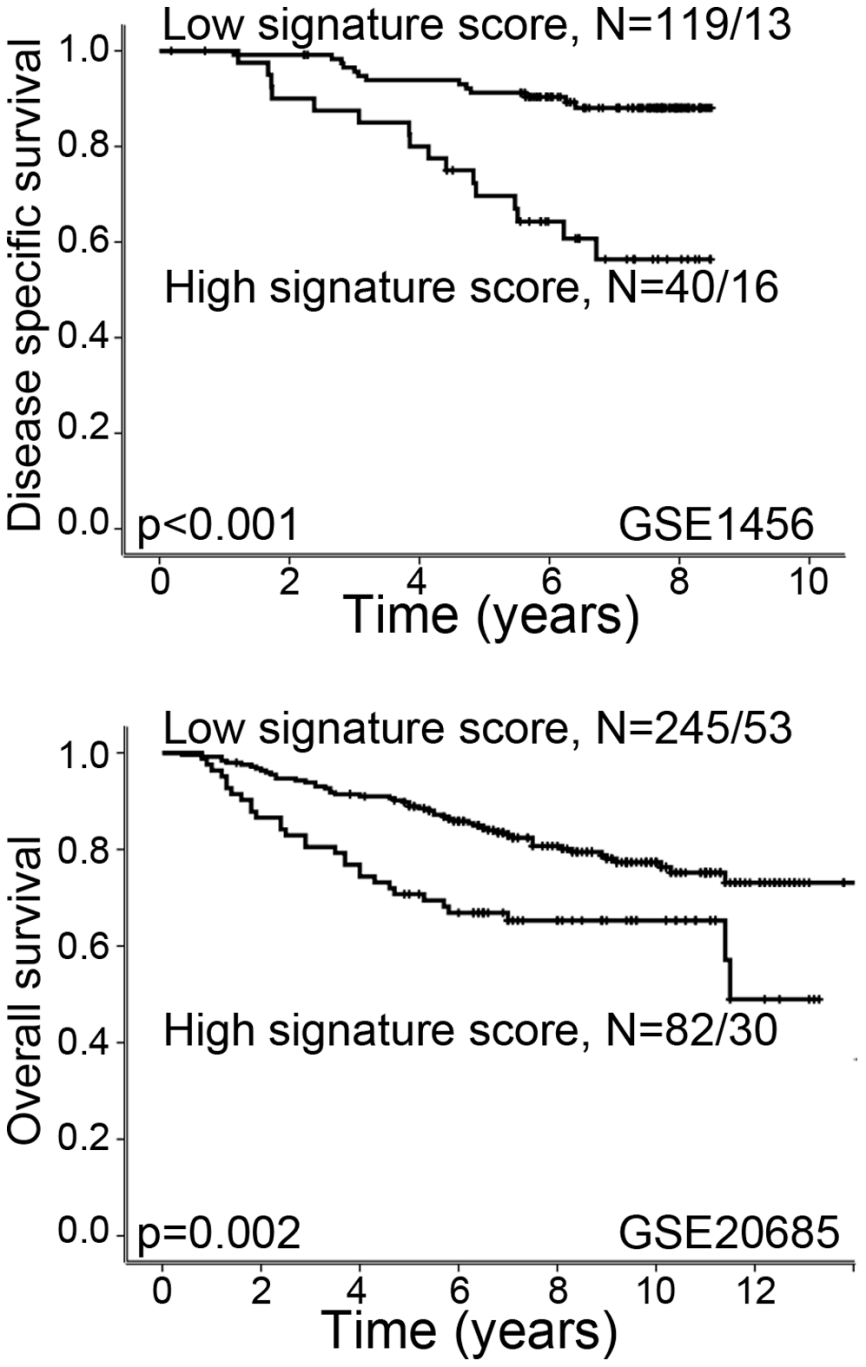
High Vascular Invasion Signature score is associated with reduced survival. High signature score is associated with reduced survival in datasets GSE1456 and GSE20685. Univariate survival analysis was performed by the Kaplan-Meier method (log-rank significance test). For each category, the number of cases is given followed by the number of breast cancer deaths.

**Figure 3:**
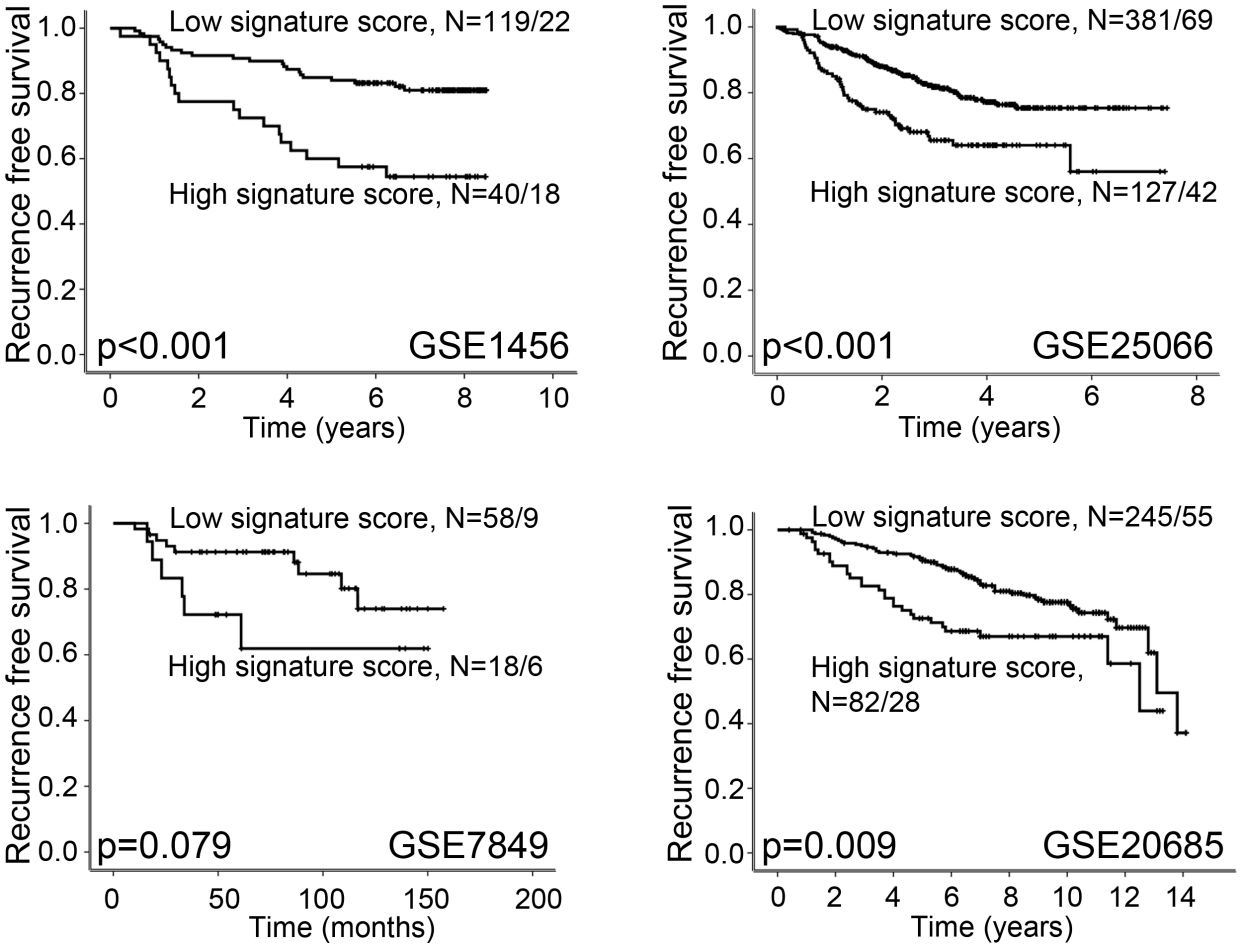
High Vascular Invasion Signature score is associated with reduced recurrence free survival. High signature score is associated with reduced recurrence free survival in data sets GSE1456, GSE2506 and GSE20685. In data set GSE7849, there is a trend between high signature score and reduced recurrence free survival. Survival curves are estimated by the Kaplan-Meier method (log-rank significance test). For each category, the number of cases is given followed by the number of breast cancer deaths.

Data set GSE1456 had information on breast cancer specific deaths and data set GSE20685 had information on overall survival [7], [12]. A high signature score was significantly associated with reduced survival in both these data sets (Kaplan-Meier method, log-rank test, p<0.001 and p = 0.002; **Figure 2**). Also, four data sets had information on recurrence free survival [7], [11], [12], [16]. A high signature score was associated with reduced recurrence free survival in three data sets, whereas the fourth data set showed a trend between reduced recurrence free survival and high signature score (p = 0.079) (**Figure 3**).

Two data sets included ER-positive patients treated with tamoxifen [16], [17]. None of these data sets showed a significant association between high signature score and probability of recurrence (data not shown). In data set GSE25066, among patients with ER positive tumors, high vascular invasion score was significantly associated with reduced recurrence free survival, p = 0.03 (data not shown). In data set GSE7849, no such association was found (data not shown).

By multivariate survival analysis, using data sets with patient survival (GSE1456 and GSE20685) or recurrence-free survival (GSE1456, GSE20685, GSE25066 and GSE7849), selected standard clinico-pathologic and molecular variables were included together with the vascular invasion signature score (**Table 5**). Initially, the vascular invasion score, histologic grade and molecular subtype were included for data set GSE1456, and vascular invasion score and molecular subtype were included for data set GSE20685. Final models showed high vascular invasion signature score to be an independent prognostic marker for decreased survival, with Hazard ratio (HR) of 2.7, p = 0.019, in data set GSE1456. For data set GSE20685, high vascular invasion score showed a HR of 2.0, p = 0.002, for reduced survival (**Table 5**).

**Table 5:** Multivariate survival analysis (Cox' proportional hazards regression model) of the vascular invasion signature score.

Data set	Variables	HR^1^	95% CI^2^	P-value^3^
**Disease specific survival**				
GSE1456^4^	Vascular invasion score	2.7	1.2–6.1	0.019
	Tumor grade	1.9	1.0–3.9	0.068
**Overall survival**				
GSE20685^5^	Vascular invasion score	2.0	1.3–3.2	0.002
**Recurrence free survival**				
GSE1456^4^	Vascular invasion score	1.9	1.0–3.9	0.063
	Tumor grade	1.8	1.1–3.1	0.032
GSE20685^5^	Vascular invasion score	1.8	1.2–2.9	0.010

1 Adjusted Hazard ratio,

2 95% confidence interval,

3 Lratio test, Final model after inclusion of: ^4^Vascular invasion score, histologic grade and molecular subtype or ^5^Vascular invasion score and molecular subtype.

Data presented for disease specific survival, overall survival and recurrence free survival.

For recurrence free survival, data set GSE1456 showed a borderline significance for the signature score, with HR of 1.9, p = 0.063, and in data set GSE20685 vascular invasion signature score is an independent prognostic marker for recurrence free survival with HR = 1.8, p = 0.01. For data sets GSE25066 and GSE7849, the vascular invasion signature was not an independent prognostic factor for recurrence-free survival (data not shown). Since Oncotype DX recurrence score predicts the risk of recurrent disease in breast cancer, this signature was included in multivariate survival analysis in the two data sets where vascular invasion score was a prognostic marker for recurrence free survival. The vascular invasion score still remained an independent prognostic marker for recurrence free survival in data set GSE1456, while in data set GSE20685, Oncotype DX recurrence signature score was an independent prognostic marker, HR = 1.8, p = 0.001 (data not shown).

### Correlation to treatment response

*GSE20194*: In 230 patients with 6 months of preoperative chemotherapy (paclitaxel, 5-fluorouracil, cyclophosphamide and doxorubicin) followed by surgical tumor resection [9], a high vascular invasion signature score showed strong correlation with pathological complete response (pCR) (p<0.001; **Table 6**).

**Table 6:** Association between the 18-gene signature score and response to treatment (mean signature score is given).

	Response					
**GSE22358 (N = 154)**	**Neoadjuvant chemotherapy**			**Chemotherapy + trastuzumab**		
	npCR^1^/pCR^2^	NR^3^/PR^4^	p-value^5^	npCR/pCR	NR/PR	p-value^5^
	3.25	−1.15	0.017	3.17	−0.12	0.089
**GSE20271 (N = 178)**	**FAC treated**			**T/FAC treated**		
	pCR	RD^6^	p-value	pCR	RD	p-value
	0.10	0.21	NS	−0.26	−0.02	NS
**GSE 20194 (N = 230)**	**Neoadjuvant chemotherapy**					
	pCR	RD	p-value			
	2.18	−0.55	<0.001			

1 Near-complete pathologic response,

2 Pathologic complete response,

3 No response,

4 Partial respons,

5 Mann-Whitney U test,

6 Residual disease.

#### GSE22358

154 women received either neoadjuvant chemotherapy alone or chemotherapy in combination with trastuzumab [15]. Among patients receiving chemotherapy only, a high signature score showed a significant association with treatment response (p = 0.017). Patients receiving chemotherapy plus trastuzumab showed a borderline significant relation between near complete or complete response and high signature score (p = 0.089) (**Table 6**).

#### GSE20271

273 patients were randomly given either weekly paclitaxel ×12 followed by fluorouracil, doxorubicin, and cyclophosphamide × 4 (T/FAC), or alone FAC×6 as neoadjuvant chemotherapy [8]. Of the 273 patients, 178 patients remained for final analysis. Response to the treatment options FAC or T/FAC-treated patients showed no correlation to the 18-gene signature (**Table 6**).

### Increased vascular invasion score is associated with other tumor progression signatures

**Figure 4:**
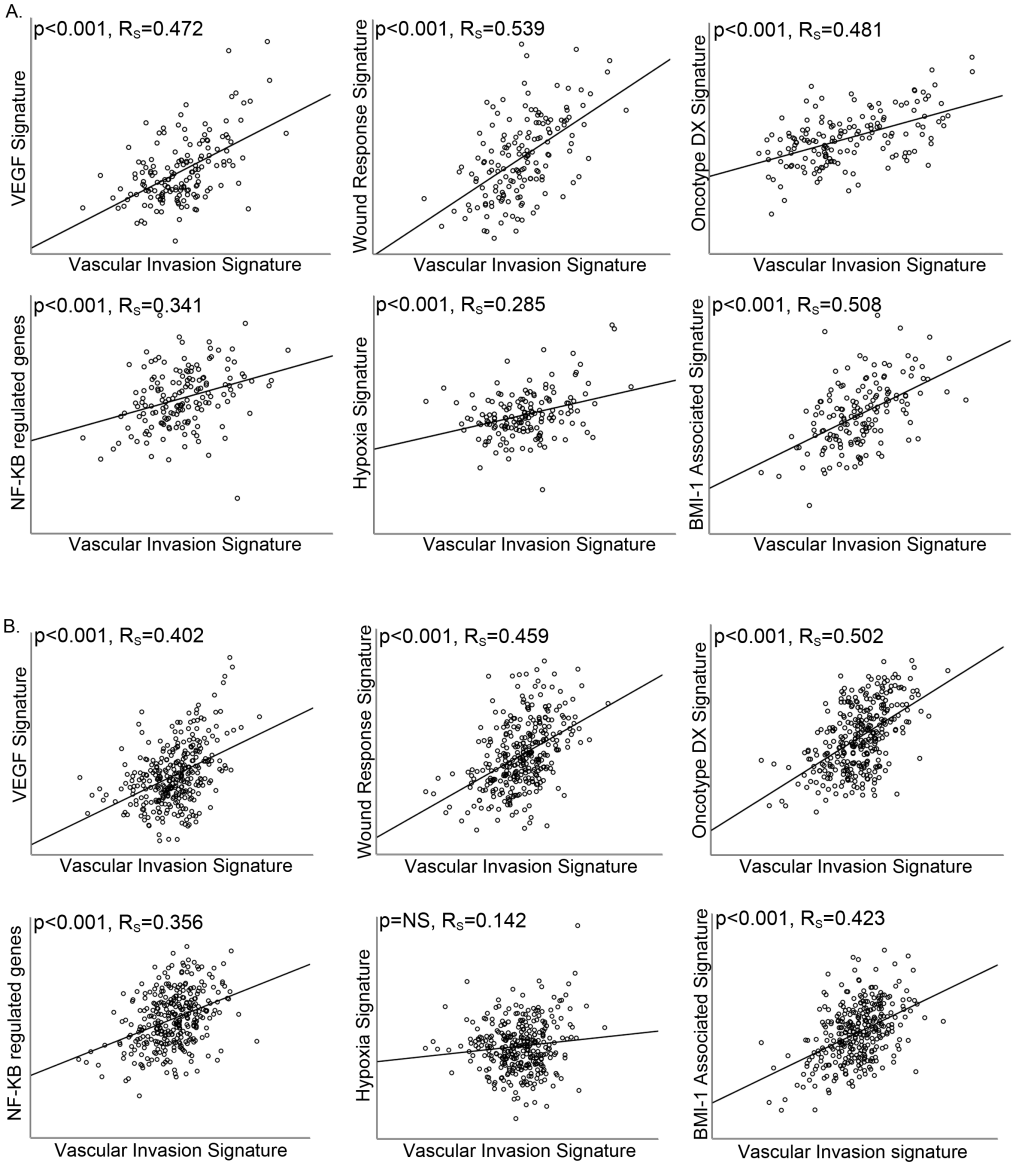
Correlation between the Vascular Invasion Signature and tumor progression signatures. Vascular Invasion Signature score shows a correlation to the VEGF signature, wound response signature, NF-Kβ related genes, hypoxia gene signature, BMI-1 signature and Oncotype DX Recurrence Score in breast cancer data sets; (A) GSE1456 and (B) GSE20685. The Spearman rank correlation test was used for bivariate correlations.

Six gene signatures related to tumor progression were mapped in the two breast cancer data sets with survival information for cancer specific death and overall survival (GSE1456 and GSE20685), and the correlations between the signature scores were explored. In data set GSE1456 (**Figure 4A**), all signatures were significantly correlated to the vascular invasion score, with Rs from 0.29–0.54. In data set GSE20685 (**Figure 4B**), all signatures except the hypoxia score show significant correlation to the vascular invasion score, with Rs from 0.36–0.50.

## Discussion

Vascular invasion is a key hallmark of aggressive malignant tumors and is considered an early marker of metastatic spread through the lymphatic or blood vascular networks. In a previous study of endometrial cancer [5], an 18-gene expression signature was established by supervised strategy based on a correlation with microscopic findings of tumor cells entering vascular structures within the tumors. By further characterization of this vascular invasion signature, expression motifs of vascular and matrix biology were found, and the signature was associated with reduced patient survival.

Since the vascular invasion signature appeared to capture important features of aggressive tumors related to tumor-microenvironment interactions, we asked whether the signature could be of value in tumor types separate from those originally studied. Here, in a study including 11 publicly available data sets of breast cancer and information on altogether 2423 patients, we found that the 18-gene vascular invasion signature showed strong associations with features of aggressive breast cancer such as high tumor grade, hormone receptor negativity, HER2 positive tumors, presence of a basal-like phenotype, reduced patient survival and response to neoadjuvant chemotherapy. This association pattern was found in most data sets studied.

However, the small data set GSE7849 did not show significant correlations between the vascular invasion signature score and ER status as well as recurrence free survival. This data set contains a low number of patients with early stage breast cancer. Differences in selection and patient characteristics, in addition to lack of power, might in part explain these negative findings.

Further, a significant association between HER2 and the vascular invasion score was only seen in two of five data sets, whereas ER and PR were associated with the signature in almost all cohorts. Interestingly, HER2 positive breast cancers appear to represent the subgroup with highest frequency of vascular invasion by tumor cells as determined on tissue sections [25]. This could in part explain the lack of significant differences in some series.

We also investigated the vascular invasion signature in three data sets with information on response to treatment. The results were not entirely conclusive, although two of the data sets, including patients treated by neoadjuvant chemotherapy, showed high signature scores in correlation with response.

Our findings support that the 18-gene vascular invasion score reflects tumor-vascular interactions and angiogenesis, by significant associations with gene signatures for VEGF-expression, the wound-response process, NF-κB and tumor hypoxia. In addition, the association with a BMI-1 related signature might indicate a relation with stem cell phenotypes.

The Oncotype DX recurrence score predicts response to chemotherapy and risk of distant recurrence in women with node negative or node positive, ER-positive breast cancer [24], [26]. The correlation between our vascular invasion signature score and the Oncotype DX recurrence score further validates that our signature identifies aggressive breast cancers. In multivariate survival analysis, the Oncotype DX recurrence score was included when examining the two data sets where the vascular invasion signature score was an independent prognostic factor for recurrence free survival. In one of these data sets, the vascular signature score maintained an independent association with prognosis, while in the other data set, Oncotype DX was the independent prognostic factor. This might indicate that both signatures capture aggressive tumor subgroups without being completely overlapping. Of note, in this study we investigated Oncotype DX cancer related genes by microarray based data, whereas the approved Oncotype DX test is performed by RT-PCR, hence it is difficult to directly compare the two signature scores.

In an independent experimental study of luminal-like and basal-like breast cancer xenograft models, basal-like tumors consistently showed significantly higher baseline scores of the 18-gene vascular invasion signature, when compared with luminal-like tumors [27]. While no clear associations between the vascular invasion score and treatment response were observed for the basal-like model, significantly higher scores were observed for luminal-like tumors treated with doxorubicin. Interestingly, this result suggests that vascular invasion could be paradoxically increased or selected for in the doxorubicin treated luminal-like tumors [27].

In conclusion, an 18-gene vascular invasion signature showed strong and consistent associations with aggressive features of breast cancer. Our results indicate that this vascular invasion score might reflect important biological characteristics involved in aggressive tumors, probably related to vascular and matrix biology. The practical value of this biomarker, in breast cancer and other tumor types, should be further studied.
